# Investigation of Crack Propagation in Locally Thermal-Treated Cast Iron

**DOI:** 10.3390/ma18020321

**Published:** 2025-01-13

**Authors:** Ona Lukoševičienė, Mindaugas Leonavičius, Vaidas Lukoševičius, Žilvinas Bazaras

**Affiliations:** 1 Department of Applied Mechanics, Faculty of Civil Engineering, Vilnius Gediminas Technical University, 10223 Vilnius, Lithuania; mindaugas.leonavicius@vilniustech.lt; 2 Department of Transport Engineering, Faculty of Mechanical Engineering and Design, Kaunas University of Technology, 44249 Kaunas, Lithuania; zilvinas.bazaras@ktu.lt

**Keywords:** austempered cast iron, crack propagation, cyclic failure, fracture, residual stresses

## Abstract

Cyclic failure problems in layered ductile iron are evident in a wide range of elements in transportation and mining equipment and depend on production technology and operating conditions. The aim of this study was to analyze the effect of residual stresses on the behavior of cyclic and static failure. The stress intensity factor, crack initiation, propagation patterns, static tension diagrams, and fracture behavior of compact tension (CT) specimens were determined. The samples used in this study were made from base cast iron, some of which were subjected to a special localized heat treatment. Experimental and analytical methods were used to conduct this study. The experiments were performed using original testing methods that adhered to the American Society for Testing and Materials (ASTM) regulations. The deformations of the partially heat-treated specimens due to residual stresses were determined using the grid method. The limiting stress intensity coefficient and the failure threshold under cyclic loading were determined in accordance with ASTM recommendations for various crack depths and openings. The results show that the heat treatment process readily produces residual stresses of different magnitudes, stress redistribution, different structures, and layer positions. Residual stresses affect the crack initiation and propagation. The stress intensity factor depends on the depth of the crack, the position of the layers, and the magnitude of the residual stresses.

## 1. Introduction

Increasing the durability and reliability of the equipment, machinery, and building structures in the power, chemical, transportation, and mining industries involves many factors that influence limit states and determine the strength of individual components and overall structures. Failure of this type of equipment can result in plant shutdowns, catastrophic failures, and significant property damage. Failures that continue to occur are proof that the design, production, and maintenance methods for many facilities are not ideal. In operation, structural and technological heterogeneity in large components leads to the development of fatigue cracks and has a significant influence on their propagation, leading to complete failure. It is essential to ensure the strength of each element in the design and operation of potentially hazardous installations. This can be achieved by using material properties in a variety of ways when determining or adjusting the capacity of an installation [1,2,3]. The equipment and service elements used in different production industries are made of materials with good mechanical properties, including strength, plasticity, break resistance, fatigue, and wear properties [4,5,6].

To obtain such properties, a special thermal treatment method is used for the processing of cast iron to form the required microstructure of the metallic substrate: ferritic, ferritic–pearlitic, ferritic–ferritic, peritic–martensitic, martensitic, martensitic tempered, and bezinitic. Ductile iron that has been subjected to the austempering process [5,7,8,9] has distinctive properties. It is often identified in other grades of cast iron as austempered ductile cast iron (ADI) [10,11,12,13,14,15,16,17].

**Figure 1:**
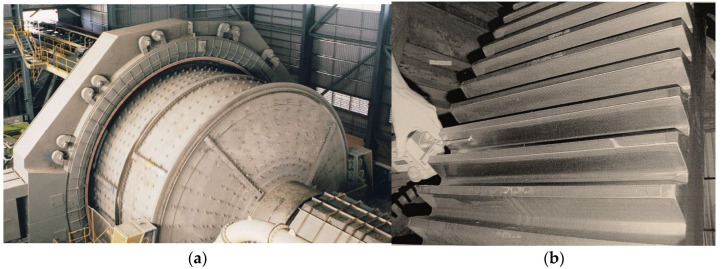
Fragment of one of the largest mineral mills (diameter more than 12 m) (**a**); the gear wheel of the mineral mill (**b**) [19].

In the manufacturing of large gears for the transport industry and for the mining of minerals, layered ductile cast iron is often used. In large and costly mining plants, individual structural assemblies and components receive special attention during the design phase or during operation. This is because they determine the performance of the whole structure and downtime for repairs is very costly. The use of ductile iron reduces the weight of the structure and ensures durability and reliability. Modern mineral mills used in mining have drum diameters exceeding 12 m and gross weights of 3800 t (Figure 1a) [18,19]. The structural elements of the mineral mills are made of ductile cast iron (body and supporting structural components) and austempered ductile cast iron alloy (e.g., transmission gears, Figure 1b) [19]. The required durability of the structures (more than 25 years) can only be achieved by using the latest technologies in design, manufacture, and operation [20,21,22,23,24,25,26].

Cyclic and dynamic loading, among other factors, influence the durability of structural elements. When considering cyclic load resistance, crack initiation and arrest are important indicators determined by the heterogeneity of the material. The heterogeneity of the material and the residual stresses develop during and remain after the production of the structural element, while the residual stresses may increase during the production process of the elements. The strength of large-sized structural elements is largely influenced by the stress state that develops during the thermal treatment process. Various studies [27,28,29,30,31,32,33] investigate the resistance of austempered iron gears to cyclic loading. Gears that are subject to cyclic loading above 10^8^ cycles should be considered. However, the existing technological capabilities to increase their resistance fail to provide the required outcome. Defects in materials and structures influence the fracture process significantly. The greatest influence is characteristic of the cracks and residual stresses, which appear after a certain service period [34,35].

Residual stresses have a significant influence on the cyclic fracture process. These stresses can be identified using different methods [36,37,38]. Residual stresses are difficult to determine using non-destructive testing methods [39,40,41]. The effects of residual stresses are constant or short-term and promote the fracture process to a larger or lesser extent at individual stages of the structure’s life, with their magnitude changing over time [42].

Residual stresses cover various components of a part, appearing on smooth surfaces and in the concentrator environment [43]. Macrostresses involve large volumes comparable to the dimensions of the part. Microstresses occur at the level of individual crystallites and influence the adjacent crystals [44]. Lattice distortions in the process of crystal formation lead to the alteration in the parameters and physic mechanical properties of the crystal [45].

Residual stresses of various magnitudes alter the indicators of a material’s mechanical properties and its resistance to cyclic loads. Residual compressive stresses resulting from plastic deformation of the surface of a part increase the durability of the part (up to 30% for smooth surface specimens and up to 60% for specimens with a concentrator). Plastic deformation alters the surface microgeometry and structure. The resulting compressive stresses alter the cumulative effect of stresses during operation, unload the surface layers of the part, and reduce the maximum stresses. Residual stresses may also cause a negative influence during operation. Cyclic loads may cause premature fracture due to cumulative external and residual effects. The same is observed if the residual compressive stresses reach the yield strength, as a result of which the fracture process is stimulated via the action of cyclic loads. In this case, stresses exceeding the yield strength lead to an increase in the plastic region and residual stresses in the compression region of the localized zones of the part (variable sign loading). These stresses lead to the development of residual tensile stresses that reduce durability under cyclic loading [46].

Residual stresses in castings may cause triaxle stress states. The stress concentration is limited due to the restriction of the plastic displacement and increased probability of crack initiation. The stress state on a surface may be biaxial, and the stresses relax in the surface layers; as a result, cracks may not always appear on the surface. Hidden internal defects are very dangerous during operation [47].

**Figure 2:**
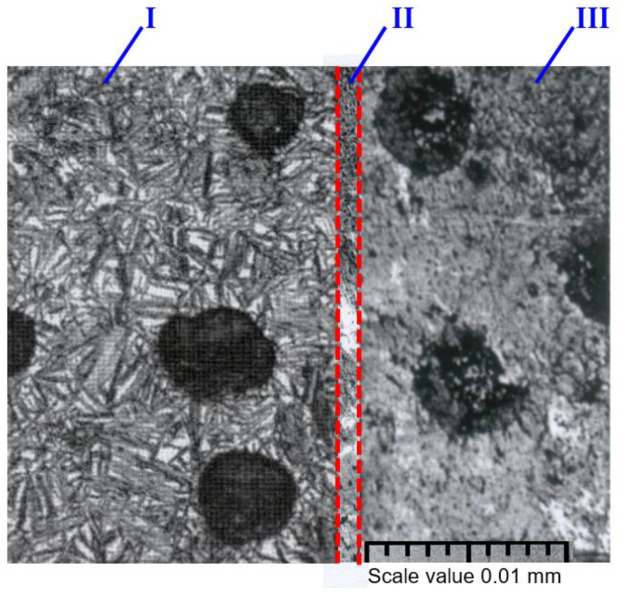
Structures of CT specimens: I—tempered layer (martensite, spheroidal graphite, ~28–36 mm); II—intermediate layer (martensite, trostite, ferrite, perlite, and graphite, ~4 mm); and III—mild layer (perlite, ferrite, graphite, ~10–15 mm) [48].

In the study reported in [48], the thermal treatment process to obtain a locally austempered ductile iron (LADI) microstructure is described. Using controlled induction technology, a part of the workpiece is heated to a temperature slightly below the melting point and maintained at that temperature for a period of time sufficient to form austenite with a uniform carbon content. After the austenitization treatment, the workpiece is cooled to a temperature in the range of 205–2300 °C. The workpiece is maintained at this temperature until a layer with an austenite microstructure is formed. The structural characteristics of the specimens are shown in Figure 2.

The study reported in [48] should be noted, as it is dedicated to investigating the cyclic fracture process of partially thermal-treated CT specimens. The study presents the results of an experimental and analytical investigation of high cycle fatigue of special cast and normalized iron. The conditions of crack arrest as the result of the action of residual stresses were examined during the investigation. By utilizing the results obtained with the help of stress intensity factor *K* and performing additional investigations, this article examines crack openings and their influence on the fracture process in view of the properties of the layer formed via thermal treatment, crack depth, etc. Additionally, in accordance with the same methodology, experimental analytical investigations were performed on CT specimens made from the base metal, i.e., cast iron, without partial thermal treatment. The obtained results were then compared with each other from the crack initiation to the complete fracture. Residual stresses also occur under the influence of an aggressive environment. A non-homogeneous stress field leads to an increase in the number of pitting corrosion foci. Residual tensile stresses promote crack propagation under the action of corrosion. Moreover, residual stresses also lead to diffusion processes in parts operated at high temperatures. Meanwhile, decrystallization processes promote material weakening factors.

A review of the research presented showed that the influence of residual stresses on the cyclic failure of cast iron has not been adequately investigated. In the manufacturing process of heavy gauge components, there are inevitable heterogeneities in cast iron materials, various defects, non-uniformity of mechanical behavior, and residual stresses. Moreover, the influences of such factors on cyclic fatigue failure are difficult to assess with current calculation methods. Therefore, it is important to identify the factors influencing the cyclic failure process of layered ductile iron, the crack propagation factors, and patterns under cyclic loading.

The contributions of this study are as follows:

(1) Cyclic strength and failure problems in a wide range of components of transport and mining equipment, depending on manufacturing technology and operating conditions, are reviewed; (2) factors affecting the cyclic failure of ductile layered cast iron are examined; (3) crack propagation factors and behavior under cyclic loading are determined; (4) the stress intensity factor and crack opening as a function of the number of loading cycles and the size of the crack is considered; and (5) the effect of residual stresses on crack initiation and propagation under static loading is determined.

This article is structured as follows:

- In Section 1, we provide the introduction and a review of methods available in the literature;
- Section 2 describes the equipment and methodology for cycle fatigue and static experiments;
- The experimental analytical results obtained are discussed in Section 3;
- The discussion is provided in Section 4;
- Finally, the conclusions are presented in Section 5.

## 2. Materials and Methods

**Figure 3:**
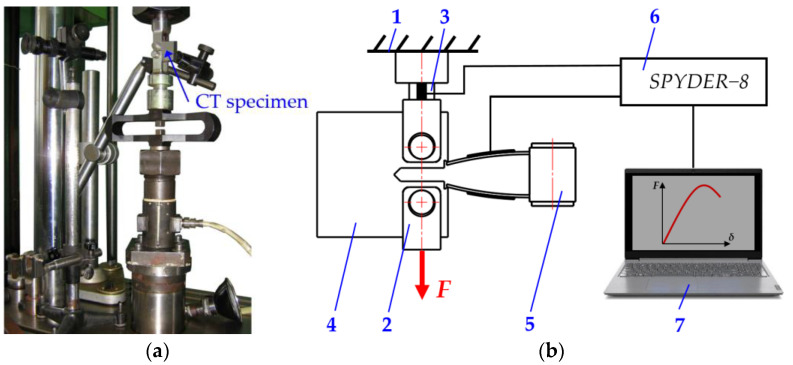
CT specimen fixture on the TIRA test 2300 machine (**a**) and the experiment scheme (**b**): 1—the body of the testing machine; 2—grip; 3—dynamometer; 4—compact tension specimen; 5—crack opening strain gauge; 6—computer metering system Spyder-8; and 7—computer.

Experimental studies were carried out to determine the static and cyclic failure performance of CT specimens at the accredited Strength Mechanics Science Laboratory of Vilnius Gediminas Technical University (VILNIUS TECH, Vilnius, Lithuania). The specimens required for the investigation were obtained from the international corporation Metso Minerals Inc. (Cannonsburg, PA, USA). In addition, specific test methods and equipment were developed for use in the experiments. The self-designed fixture with a CT specimen was fitted to the TIRA test machine TIRA test 2300, max. load 100 kN (TIRA GMBH, Schalkau, Germany), with an electronic part, and then subjected to the axial load (Figure 3a).

Furthermore, the following measurement tools were used: strain gauges to measure crack openings, DDS-10-1 (base 10 mm)—±1.5% accuracy; dynamometer (in the TIRA test 2300)—±1.5% accuracy; computer metering system with analog-to-digital converter SPIDER-8; data registration and processing computer program CATMAN EXPRESS 3.0; and tool microscope BM1-1C, with magnification up to ×50.

The tests were carried out in accordance with the following standards and regulations: ASTM E399-90 [49]; ASTM E647 [50]. During the failure test of the compact specimen, the magnitude of the force, the number of cycles, the opening, and the size of the crack are observed.

Changes in specimen shape are detected by measuring the opening of the crack at the edges of the specimen using a strain gauge sensor. The measurement system records the force F and the opening δ and a correlation diagram is displayed on the monitor. An electrical current signal proportional to the magnitude of the force is obtained from the dynamometer, and a signal proportional to the opening of the crack is obtained using a special strain gauge. As the crack opens, the distance between the edges of the notch in the specimen increases, which is measured using elastic strain gauge elements with strain gauge sensors bonded to them. It can be seen from Figure 3b that the tips of the tensile elements sit at the ends of the notch in the specimen and move away from each other with the specimen. The tests were carried out at a frequency of 15–20 Hz. Periodic operation of the test machine involves checking the parameters of the load cycle on the oscillograph display and on the control panel. The tests were carried out at room temperature. In addition, ultrasonic, magnetic fluorescence, and dye trace methods were applied to clarify the shape of the crack front.

**Figure 4:**
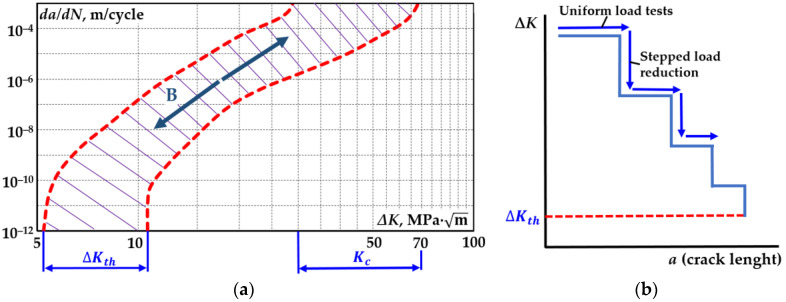
Diagram showing the direction of load variation for the determination of the fracture threshold (**a**); ΔK reduction test scheme (**b**).

A pre-crack is grown in the specimen prior to the threshold measurement procedure. This is to reduce the influence of the notch as a stress concentrator and to produce a uniform fatigue crack. The compact specimen pre-crack is formed using the fatigue test. Figure 4a shows the direction of variation in the test load to determine the rate of deterioration. The fracture toughness evaluation procedure starts by loading the specimen under the conditions at which the pre-crack grew, with a further reduction in the loading force ΔF (simultaneously reducing the range of the stress intensity factor ΔK). This test method is indicated by the B arrows in Figure 4b.

This is applied until a value of ΔK is obtained at the required fatigue crack growth rate da/dN. The ΔF step size must not exceed 10% as the force is progressively reduced and the gap growth between these steps should not be less than 0.50 mm. The ΔK reduction test scheme is shown in Figure 4b. As the threshold is approached, the force changes become very small. At the microscopic level, the fatigue crack is known to propagate in spikes. The size of the spike represents only a few parameters of the crystal lattice. This step growth is associated with a loss of plasticity and the appearance of small cracks in the crack tip environment. When the number of small cracks reaches a critical number, the crack growth jumps. The fracture threshold is determined using a force value at which the fracture velocity is no longer recorded. The arrows indicate the direction in which the load is changed by setting the parameters ${\Delta K}_{th}$ and $K_{c}$. If ${\Delta K}_{th}$ is required to calculate the cyclic strength, then only a plot of the crack growth rate versus the range of the stress intensity factor is produced (part of the kinetic diagram). After the determination of ${\Delta K}_{th}$ (Figure 4a, loading arrow B), a CT specimen with a crack was used to determine the fracture toughness using static testing (Figure 4a, loading arrow A).

The cyclic experiment was carried out at the asymmetry coefficient of the stress cycle as follows:

(1) $$r=\frac{F_{min}}{F_{max}}$$

The change in force variation, $\Delta F=F_{max}-F_{min}$, is the interval between the maximum $F_{max}$ and minimum $F_{min}$ forces per loading cycle and the change in stress intensity factor, $\Delta K=K_{max}-K_{min}$, is the interval between the maximum $K_{max}$ and the smallest $K_{min}$ values of the stress intensity factors. The minimum stress intensity factor, $K_{min}$, is the minimum value of the stress intensity factors per cycle. This value corresponds to $F_{min}$ at r>0 and is assumed to be 0 at r≤0.

Due to the complexity of the thermal treatment process, structural changes were observed in individual parts of the specimens. The microstructure of the thermal-treated part consisted of coarse needle-like martensite, globular graphite, single inclusions of cementite, and residual austenite. The microstructure of the specimen part that was not subjected to the thermal treatment consisted of perlite, ferrite, and graphite. The structure of the intermediate layer consisted of trostite, ferrite, perlite, and spheroidal graphite. Some specimens had cementite inclusions. The resulting microstructure of the thermal-treated part was typical of austempered cast iron. In the thermal treatment, the mechanical properties of the mild layer changed slightly compared with the properties of the base metal. The microstructure of base cast iron consists of perlite with a small amount of ferrite and spherical graphite.

**Figure 5:**
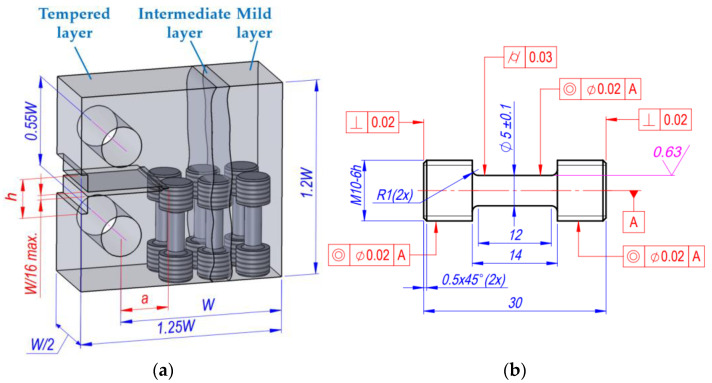
The main dimensions of the CT specimens (h—notch height, a—crack length) for fracture testing experiment (**a**); shape and dimensions of the specimens for monotonous tensile experiments (**b**) (units in mm).

Following the partial thermal treatment, residual stresses developed in individual parts of the CT specimens due to the structural change, which affects the fatigue failure process. Following the thermal treatment, the hard layer remained tensioned, and the mild layer was subjected to compression. An intermediate layer of various dimensions was formed between the hard and mild layers. As a result, residual stresses developed. The main dimensions of the CT specimens are presented in Figure 5a.

Five specimens made of cast iron were selected to study the influence of residual stresses and mechanical properties. All specimens were mechanically processed to provide the standard dimensions of CT specimens (62.5 × 60 × 25 mm) according to the ASTM [48] requirements (Figure 5a). Two CT specimens (B1, B2) meeting the ASTM requirements were made from base cast iron for determination of resistance to cyclic loading and fracture indicators. During the next step, a standard notch geometry was made. In the formation of the notch and holes, the tension was partially relaxed, and the stress distribution changed. The residual compressive stresses began to act on the hard layer and the notch height changed. In the intermediate layer and the mild layer, the residual stresses also changed.

The measurement of the dimension of the height of the notch was performed at 12 mm and the results are presented in Table 1. In all specimens, height h decreased to $h_{1}$.

To determine the mechanical properties of the materials, we selected specimens from which cylindrical tensile specimens were produced following cyclic testing (Figure 5b). The main task of an experimental device is to provide a homogeneous deformation state to the specimen to be deformed. This is achieved by the high coaxiality of the grippers and the rigidity of the machine. Samples used for tension tests with a linear stress state must provide a homogeneous stress state in the test section of the test specimen. Figure 4b shows a drawing of the specimen that best satisfies these conditions. The tensile tests were performed according to ISO 6892-1:2016 [51]. Mechanical characteristics were measured with an error that did not exceed ±1% of the deformation scale. The following mechanical properties of individual layers were determined (Table 2).

**Figure 6:**
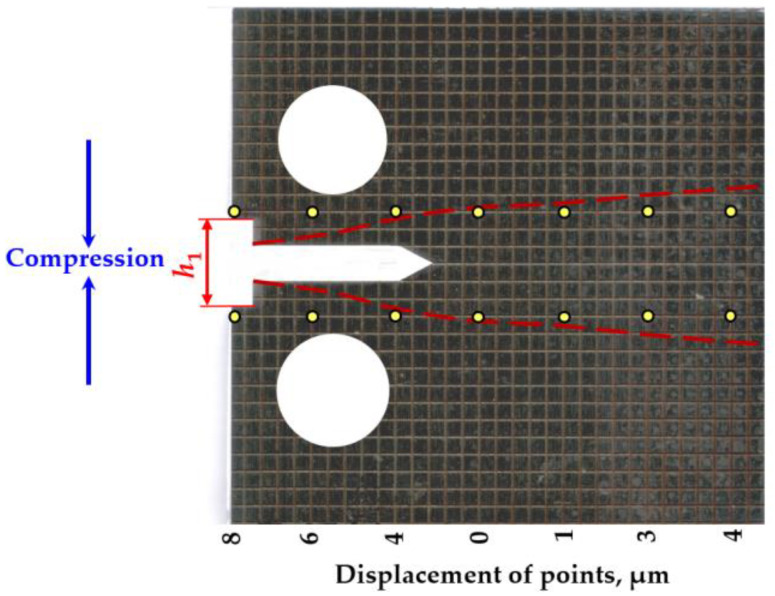
Distribution of displacements and stresses in the H3 type specimen.

A special example (H3) (Figure 6) of partially thermal-treated specimens was prepared to investigate the deformation of the CT specimens. The preparation of the specimen consisted of several stages. The H3 specimen was made according to the dimensions of the previously described specimens. Subsequently, a grid (2 × 2 mm) was made on the side surface of the specimen using a laser. Next, a measuring notch was made and the distance between individual points was measured. The notch and holes were then cut according to ASTM [49] and the variation between the same points was measured. The measurement results are presented in Figure 6. Residual stresses resulted in a grid distortion that reflected the deformations perpendicular to the notch. The red dashed lines in Figure 6 show the distribution of residual stresses in the prepared specimen.

## 3. Results

The hardness of the CT specimens was measured using the Brinell method according to ASTM E10 [52]. The hardness values obtained during the test are provided in Table 3. The remaining CT specimens demonstrated comparable levels of measured hardness. This elevated hardness can be associated with the presence of martensite. The hard surface layer in particular exhibits the most favorable mechanical properties.

The different stress intensity values obtained depended on the position of the individual layers, mechanical properties, structure, depth of crack, and other factors. The crack opening was additionally measured. During the test, the crack opening was periodically measured during static loading. The crack threshold, $\Delta K_{th}$, was the asymptotic ΔK value at which da/dN approached zero. This value was considered as the limiting value of the variation interval of the stress intensity factor (cracking threshold) $\Delta K_{th}.$ The fracture threshold for the base cast iron specimens was determined: ${\Delta K}_{th}=8–10\;MPa\sqrt{m}$.

**Figure 7:**
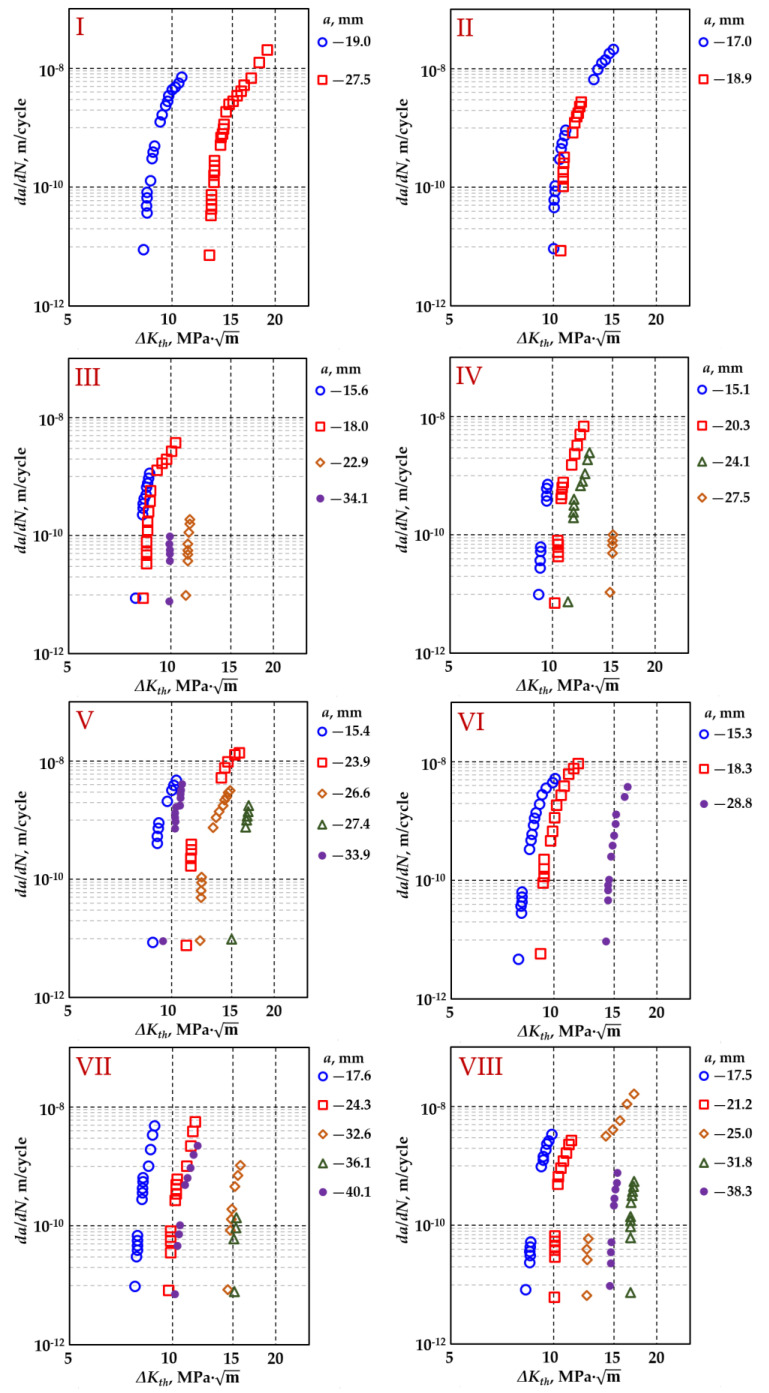
Dependence of crack growth rate on fracture threshold interval (**I**–**VIII**―specimens No).

For partially thermal-treated cast iron, the cyclic fracture process was analyzed using CT specimens. During the tests, the fracture threshold, ${\Delta K}_{th}=8–17\;MPa\sqrt{m}$, was determined for eight specimens according to [48,49] (Figure 7).

The change in stress intensity ΔK was calculated according to ASTM E647 [50] as follows:

(2) $$\Delta K=\frac{\Delta F}{B\cdot \sqrt{W}}\frac{2+\lambda }{{\left(1-\lambda \right)}^{3/2}}\left[0.886+4.64\lambda -13.32\lambda^{2}+14.72\lambda^{3}-5.6\lambda^{4}\right]$$

where λ=a/W; the formula is valid when a/W≥0.2.

**Figure 8:**
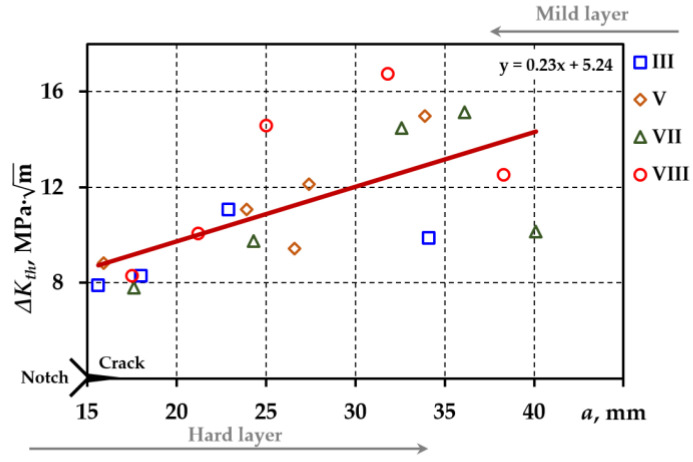
Dependence of the stress intensity factor interval on the depth of the crack: scatter plot with a regression line.

Figure 8 illustrates the generalized dependence of the stress intensity factor interval on crack depth obtained by processing the experimental data from specimens III, V, VII, and VIII.

The analysis of the relationships between the fracture threshold and the crack sizes shows that the threshold increases as the crack approaches the intermediate and mild layers and decreases as it enters the mild layer (specimens VII and VIII). The maximum magnitude of $\Delta K_{th}$ is reached just before the intermediate layer.

**Figure 9:**
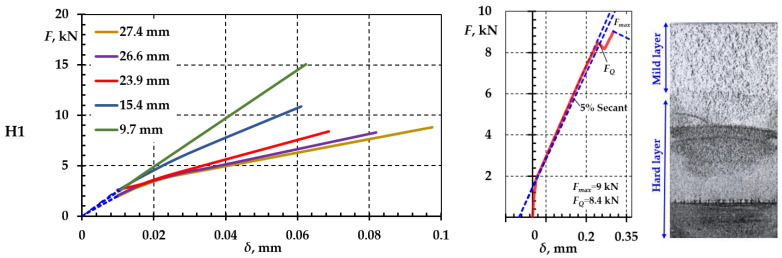

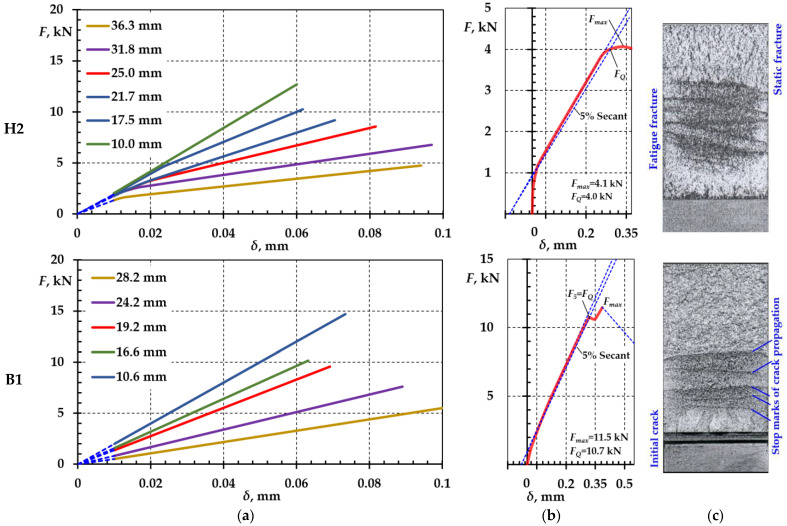
Dependence of force on the crack opening for different depths of crack (**a**); crack opening diagrams (**b**); and fractures of CT specimens (**c**).

During the experimental study, periodic static loading and measurement of the specimens with cracks were performed. Due to the complexity of the testing process, samples H1, H2, and B1 were selected for further analysis. The scheme applied to the CT specimen static fracture test is shown in Figure 3, Figure 4 and Figure 5. The resulting dependences of force value F and crack opening δ are shown in Figure 9.

For all specimens that only had the notch ≈12 mm, dependence F−δ was linear. As soon as the crack reached a>15 mm, a deviation from linearity was observed. As the crack increased, dependence F−δ was non-linear and depended on the position of the hard layer and the intermediate layer. There was a measurement uncertainty of 0.01 mm before the opening in the dependences shown in Figure 9a,b.

All specimens were compared to determine the process of cyclic fracture by applying the patterns of crack opening and propagation from crack initiation. According to the data presented above, the effect of residual stresses occurred only after the 12 mm notch was made. To open a crack, δ=0.02 mm, CT specimens had to be subjected to a force 1.1–1.25 times higher than the force that would have been applied to the base cast iron specimens.

As the crack increased under the action of cyclic loading, the value of force changed when measuring the opening and, in some cases, the difference was two-fold. The size and position of the intermediate layer in the CT specimen became available at the crack depth of 17–27 mm.

In the CT specimen made from base cast iron, the dependences F−δ were close to the straight line (Figure 9a,b). Noticeable deviations depended on the shape of the crack front and changes in the structure of individual zones. The complete fracture was obtained by breaking all CT specimens with a crack and calculating the static break and fracture parameters. Fractures of CT specimens after static breaking are shown in Figure 9c. The static breaking diagrams correspond to type II specified by the standard ASTM E647 [51].

The critical stress state at the crack tip is described by the fracture toughness KIC. The value of this parameter depends on the micro-properties of the material, which determine its fracture resistance. The fracture toughness was determined after the experiment by verifying the compliance with the basic conditions regulated by ASTM E399 [49] as follows:

(3) $$\left.\begin{array}{c} a\\ B\\ W-a \end{array}\right\}\geq 2.5\frac{{K_{Q}}^{2}}{{\sigma_{ys}}^{2}},\frac{F_{\max}}{F_{Q}}\leq 1.1,\;0.45W\leq a\leq 0.55W$$

The values of the required parameters are provided in Figure 5.

The stress intensity factor, $K_{Q}$, was calculated according to the following formula:

(4) $$K_{Q}=\frac{F_{Q}}{B\cdot W^{1/2}}f\left(a/W\right),\;\begin{array}{l} f\left(\frac{a}{W}\right)=\left[\left(2+\frac{a}{W}\right)/{\left(1-\frac{a}{W}\right)}^{3/2}\right]\times \\ \times \left(0.866+4.64\left(\frac{a}{W}\right)-13.32{\left(\frac{a}{W}\right)}^{2}+14.72{\left(\frac{a}{W}\right)}^{3}-5.6{\left(\frac{a}{W}\right)}^{4}\right) \end{array}$$

When the basic conditions are met, the stress intensity factor $K_{Q}$ is the fracture toughness indicator $K_{IC}$. The analysis of the experimental results of the CT specimens H1 and H2 and the base specimen B1 showed that a larger force was required to open a larger crack.

**Figure 10:**
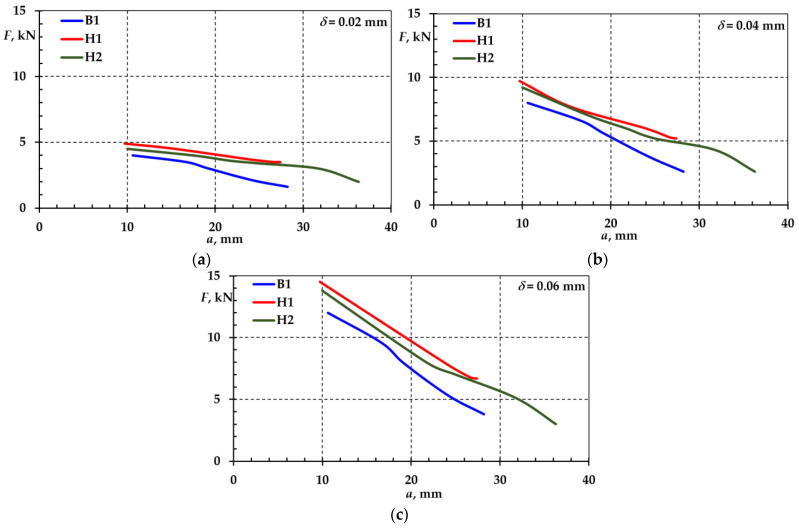
Dependence of force on crack depth and crack opening: δ=0.02 mm (**a**), δ=0.04 mm (**b**), and δ=0.06 mm (**c**).

The dependence of force variations in crack depth, a, at different openings, δ, are shown in Figure 10.

The results obtained during the experimental tests of the CT specimens H1 and H2 and the base specimen B1 were applied to the calculation of the normal stresses at the crack tip, which was performed using the ASTM standard methodology as follows:

(5) $$\sigma_{1}=\frac{F}{A}+\frac{M}{Z}=\frac{F}{B}\left[\frac{4W+2a}{{\left(W-a\right)}^{2}}\right]$$

Bending moment M was calculated using the following expression:

(6) $$M=F\cdot \left[a+\frac{W-a}{2}\right]$$

The rectangular cross-sectional area of the CT specimen (net) was calculated as follows:

(7) $$A=\left(W-a\right)\cdot B$$

The section modulus of the CT specimen was calculated as follows:

(8) $$Z=\frac{{\left(W-a\right)}^{2}\cdot B}{6}$$

**Figure 11:**
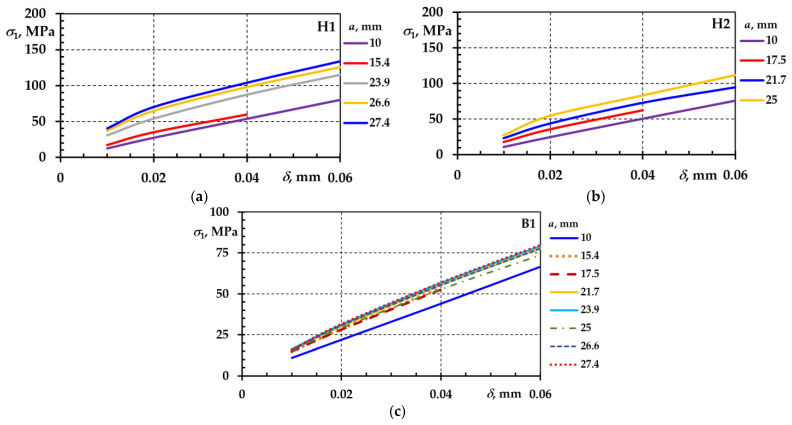
Normal stresses at the crack tip depending on crack depth a and crack opening δ in CT specimens: H1—(**a**); H2—(**b**); and B1—(**c**).

The variation in normal stresses at the crack tip depending on the crack depth and opening is presented in Figure 11. The residual stresses remained in the material even after the stress sources were removed.

Residual stresses are generally defined as deformation caused by treatment, structure, or other factors. Residual stresses alter the conditions of cyclic loading and thus the resistance to fatigue crack initiation and propagation.

Following the thermal treatment, the CT specimens developed residual stresses. During the investigation, it was noticed that deformations appeared after the formation of the notch. After accepting certain assumptions (linear law of residual stress distribution), an analysis of residual stresses and their effect on cyclic loading was conducted.

**Figure 12:**
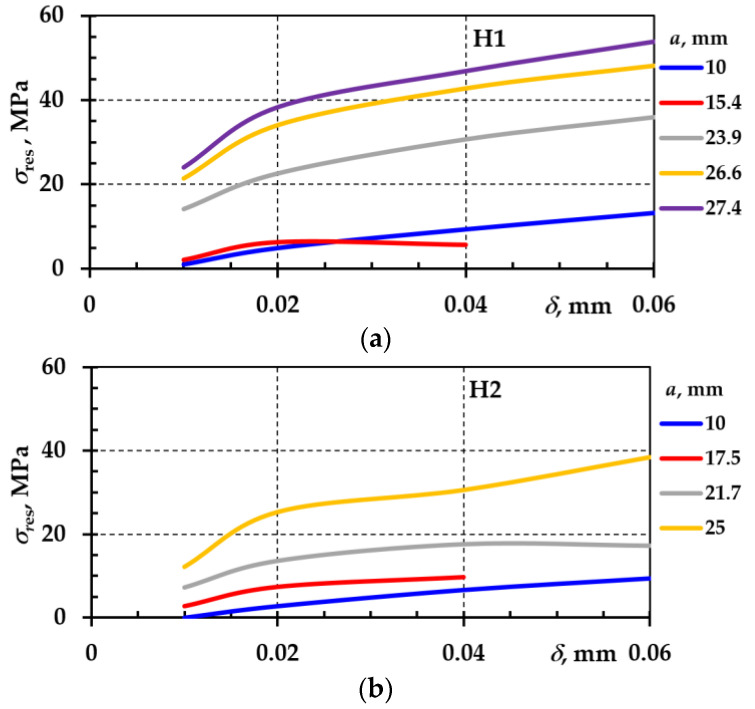
Residual stresses at the crack tip depending on crack depth *a* and opening in CT specimens: H1—(**a**); H2—(**b**).

The residual stresses were obtained by summing the normal stress values of CT specimens H1 (Figure 11a) and H2 (Figure 11b) with the normal stress values of the base specimen B1 (Figure 11c). The results are shown in Figure 12a,b.

## 4. Discussion

The presence of residual stresses in structural members can be attributed to the following factors: The application of a load exceeding the elastic limit to a member results in the emergence of residual strains within the constituent fibers. These strains persist even after the load, preventing the adjacent elastically stressed fibers of the same element from regaining their original length. Consequently, residual stresses are generated. The occurrence of residual stresses is attributed to a variety of physical, chemical, and thermomechanical factors that cause irreversible processes within the material.

It is important to distinguish between crack initiation and failure rates when considering cyclical load resistance properties. The heterogeneity of the material and the residual stresses produced during its manufacture are retained after the structural element. Furthermore, residual stresses may increase during the fabrication of the elements. The strength of large structural elements is strongly influenced by the state of stress and strain generated by the heat treatment process. Additionally, the arrest of fatigue cracks and the limit state of this process (i.e., the appropriate conditions for the crack to arrest) have a significant influence on the safe condition of the structure.

Residual stresses occur after casting, forging, rolling, stamping, heat treatment, and other post-treatment processes when heterogeneous plastic deformations, phase transformations, diffusion effects, etc., accumulate in the material. The test data show that the heat treatment process readily produces residual stresses of different magnitudes, structures, and layer positions.

Calculation methods based on fracture mechanics criteria are applied to the strength analysis of structural elements. Of particular importance is the limiting stress intensity factor ${\Delta K}_{th}$, which is determined via standardized tests using semi-natural specimens. Theoretical and experimental studies show that the stress intensity factor depends on the yield strength, the strength ratio, the limit of durability, etc. It also depends on the coefficient of cycle asymmetry, temperature, environmental effects, overloading, loading history, structural features, mechanical properties of the material, and other factors. This is due to the fact that higher average stresses lead to higher crack propagation rates.

In some applications (bending and torsion), residual deformation occurs when the stresses in the boundary layers exceed the yield stress during loading. Residual stresses influence the initiation and propagation, change the magnitude of crack opening and closing, and can inhibit or promote crack growth at the crack tip. Investigations show that the stress intensity factor depends on the depth of the crack, the position of the layer, and the magnitude of the residual stresses.

## 5. Conclusions

Three different layers were formed in the CT specimens using local thermal treatment. The microstructure of the thermal-treated part consisted of coarse needle-like martensite, globular graphite, single inclusions of cementite, and residual austenite. The microstructure of the specimen part that was not subjected to the thermal treatment consisted of pearlite, ferrite, and graphite. The structure of the intermediate layer consisted of trostite, ferrite, pearlite, and spheroidal graphite. The resulting microstructure of the thermal-treated part was typical of austempered cast iron. The microstructure of the part that was not subjected to the thermal treatment was typical of normalized cast iron. Following the thermal treatment, the hard layer remained tensioned, and the mild layer was subjected to compression. An intermediate layer of various dimensions was formed between the hard and mild layers, and thus residual stresses developed.

The main conclusions that can be drawn from this work are as follows:

1. Periodic static loading and opening measurement of the specimens with a crack showed that when the crack reached a depth of 17–27 mm, the effect of residual stresses was noticeable compared with the CT specimens from base cast iron. Noticeable differences depended on the change in the structure of individual zones of the crack front and the position of the intermediate layer.
2. The fracture threshold, ${\Delta K}_{th}$, was determined according to the adapted ASTM methodology in view of different crack depths and crack openings (passing through different layers in the CT specimens). The limiting stress intensity coefficient and the failure threshold under cyclic loading were determined in accordance with ASTM recommendations, ${\Delta K}_{th}$, for various crack depths and openings. The fracture threshold for locally heat-treated cast iron is ${\Delta K}_{th}=8–17\;MPa\sqrt{m}$ at fracture growth speeds close to $da/dN=10^{-11}$ m/cycle, whereas for basic cast iron, it is —${\Delta K}_{th}=8–10\;MPa\sqrt{m}$.
3. After mechanical processing of all workpieces in accordance with ASTM requirements, it was found that the specimens were partially relaxed, and stress redistribution occurred due to the residual compressive stresses acting on the hard layer. The grid distortion on the side of the specimens characterized the deformations and displacements in the crack propagation environment.
4. The analysis of the relationships between the fracture threshold and the crack sizes suggests that there may be a correlation between the two variables. It appears that as the crack approaches the intermediate and mild layers, the threshold may increase, while as it enters the mild layer, the threshold may decrease. Additionally, it seems that the maximum magnitude of the fracture threshold may be reached just before the intermediate layer.
5. The results of the analytical calculations demonstrate that both normal and residual stresses increased in combination with an increase in crack depth and crack opening.

## Figures and Tables

**Table 1:** Notch height measured data.

Specimen	Notch Height *h*, mm (Before)	Notch Height *h*_1_, mm (After)	Variation
H1	12.021	12.007	0.014
H2	12.029	12.023	0.006
Special H3	12.03	12.024	0.006
Base B1	12.02	–	–
Base B2	12.025	–	–

**Table 2:** Mechanical properties of the materials.

	Base Material	Hard Layer	Intermediate Layer	Mild Layer
$\sigma_{ys}$, MPa	630–634	957–963	603–608	602–607
$\sigma_{u}$, MPa	863–933	1254–1261	864–874	864–869
E, MPa	158–160	159–169	~163	163–165
ψ, %	3.7–6.7	3.6–4.1	2.8–3.2	2.9–3.3

**Table 3:** The results of the surface hardness measurement.

	CT Specimen Distance from the Left (Hard Layer) to the Right (Mild Layer)				
	5 mm	17 mm	33 mm	47 mm	59 mm
Brinell hardness, HB	388	401	401	302	285

## Data Availability

The original contributions presented in this study are included in the article. Further inquiries can be directed to the corresponding authors.

## Nomenclature

The following symbols are used in this manuscript:

Latin symbols	
a	Crack depth (mm)
A	Cross-sectional area (mm^2^)
B	Specimen width B=0.5W (mm)
da/dN	Crack growth rate (m/cycle)
E	Modulus of elasticity (MPa)
${F,F}_{5},F_{Q}$	Force (N)
ΔF	$\mathrm{The}\;\mathrm{interval}\;\mathrm{between}\;\mathrm{the}\;\mathrm{maximum}\;F_{max}$ $\mathrm{and}\;\mathrm{minimum}\;F_{min}$ forces per loading cycle (N)
$h,\;h_{1}$	Notch height (mm)
K	$\mathrm{Stress}\;\mathrm{intensity}\;\mathrm{factor}\;(\mathrm{MPa}\cdot \sqrt{m}$)
$K_{Ic}$	$\mathrm{Fracture}\;\mathrm{toughness}\;(\mathrm{MPa}\cdot \sqrt{m}$)
ΔK	$\mathrm{The}\;\mathrm{interval}\;\mathrm{between}\;\mathrm{the}\;\mathrm{maximum}\;K_{max}$ $\mathrm{and}\;\mathrm{the}\;\mathrm{smallest}\;K_{min}$$\mathrm{values}\;\mathrm{of}\;\mathrm{the}\;\mathrm{stress}\;\mathrm{intensity}\;\mathrm{factors},\;\Delta K=K_{max}-K_{min}$$,\;(\mathrm{MPa}\cdot \sqrt{m}$)
${\Delta K}_{th}$	$\mathrm{Fracture}\;\mathrm{threshold}\;(\mathrm{MPa}\cdot \sqrt{m}$)
M	Bending moment (N∙mm)
r	Asymmetry coefficient of the stress cycle
$R^{2}$	Coefficient of determination
W	Specimen length (mm)
x	Independent regression equation variable
y	Dependent regression equation variable
Z	Section modulus (mm^3^)
Greek symbols	
δ	Crack opening (mm)
λ	Coefficient, λ=a/W
$\sigma_{1}$	Normal stress (MPa)
$\sigma_{res}$	Residual stress (MPa)
$\sigma_{ys}$	Elastic limit or yield strength; the stress at which 0.2% plastic strain occurs (MPa)
$\sigma_{u}$	Ultimate stress (MPa)
ψ	Reduction in area (%)

## Abbreviations

The following abbreviations are used in this manuscript:

ADI	Austempered ductile cast iron, also known as ADI.
ASTM	American Society for Testing and Materials
B1, B2	Base cast iron specimens (not heat-treated)
CT	Compact tension specimen
H1, H2	Partially thermal-treated cast iron specimens
H3	Partially thermal-treated cast iron specimen with the grid
HB	Brinell hardness
GOST	Government standard, gosudarstvennyy standart (Russian: гoсударственный стандарт)
ISO	International Standard Organization
LADI	Locally austempered ductile cast iron, also known as LADI.
